# Fretting wear behavior of Inconel 718 alloy manufactured by DED and treated by UNSM

**DOI:** 10.1038/s41598-023-28128-8

**Published:** 2023-01-24

**Authors:** Chao Li, Ruslan Karimbaev, Shengjie Wang, Auezhan Amanov, Dagang Wang, Magd Abdel Wahab

**Affiliations:** 1 Laboratory Soete, Ghent University, Ghent, Belgium; 2 Department of Fusion Science and Technology, Sun Moon University, Asan-si, Korea; 3 Department of Mechanical Engineering, Sun Moon University, Asan-si, Korea; 4 School of Mechatronic Engineering, China University of Mining and Technology, Xuzhou, 221116 China

**Keywords:** Mechanical engineering, Mathematics and computing

## Abstract

Alloy 718 is commonly used in the maritime and aerospace industries due to its strength and durability, particularly in engine rotating components such as disks, fan blades, and high-pressure compressors. As a new type of 3D printing technology, directed energy deposition (DED) can employ lasers to melt metal powders or wires to fabricate arbitrary-shaped workpieces directly from customized data, thereby making machining more synergistic and intuitive. However, the surface properties of the DED-printed alloy 718 samples, such as surface roughness and wear resistance, are typically subpar. By introducing severe plastic deformation to the near-surface, ultrasonic nanocrystal surface modification (UNSM) can be used as a post-processing method and results in altered properties. The uniaxial tensile test reveals that the UNSM-treated alloy 718 exhibits a higher mechanical property. Moreover, using a fretting test rig in accordance with the cylinder-on-plane agreement, a higher wear resistance for UNSM-treated alloy 718 is observed. This study employs the finite element method to fully comprehend the effect of UNSM on wear performance. The fretting wear process of Inconel 718 alloy is established using an energy-based finite element model. Considering the severe practical scenarios, the Johnson–Cook constitutive model is implemented, with the linear isotropic hardening model capturing the plastic behavior. In comparison to experimental measurements, the finite element results demonstrate unprecedented wear loss consistency with an error of less than 2%. Therefore, we conclude that the finite element model built in this study exhibits a high accuracy and can be used to analyze the effect of UNSM on fretting wear behavior. According to finite element analysis, as the normal load increases, the improvement in wear resistance induced by UNSM decreases. Given that the finite element model is based on the energy method, the effects of coefficient of friction (COF) and wear coefficient modified by UNSM are investigated separately. According to the findings, the UNSM-modified COF and wear coefficient play a significant role in determining the wear characteristics. Due to the removal of a substantial amount of material from the central area of the alloy 718 surface by wear, it is also possible to observe that severe plastic strains are primarily concentrated at the edges of the wear scars.

## Introduction

On a simplified basis, superalloys are alloys containing at least one element of nickel, cobalt, or iron that exhibit high performance and excellent stability in extreme environments^1^. Due to the various elements present in each type of superalloy, custom-tailored properties and applications have been developed through years of industrial production^2–4^. One of the superalloys, namely Ni-based alloy, possesses a remarkable combination of mechanical strength and creep resistance^5^. Alloy 718 is a family of nickel-based superalloys and is considered the most commonly used^6^. Alloy 718 has a broad range of applications in the aerospace, energy (oil, gas) exploration, and power generation industries^7^. Specifically, superior mechanical performance, including a high tolerance for stress levels and temperatures (up to 1400 °C), qualifies it for use in particularly harsh environments. It is worth note that alloy 718 is used extensively in critical machinery components such as nuclear reactors and commercial and military aircraft turbine engines^8,9^. With the global energy crunch and the quest for fuel efficiency, the aerospace industry seeks to combine high mechanical performance and reliability with lightweight components^10^.

Traditionally, alloy 718 workpieces were primarily manufactured using subtractive techniques (e.g., milling, turning, and drilling), and metallurgical processes (welding, casting, and forging)^11,12^. However, it is difficult to obtain the desired complex geometries precisely because of their extraordinary mechanical properties^12^. In addition, cutting tools in conventional machining are highly wearable, resulting in excessive downtime and high costs^13^. In response to the problems encountered above, additive manufacturing (AM), has been put into production and has shown unprecedented advantages as an emerging concept of machining and forming^14^. With the development of computer-aided design (CAD) software, directed energy deposition (DED) becomes one of the most state-of-art AM techniques for metal parts^15^. DED can employ lasers to melt metal powders or wires to fabricate arbitrary-shaped products directly from customized CAD data. Compared to other AM techniques, DED makes the fabrication of alloy 718 more synergistic and intuitive, while ensuring good overall performance retention. In addition, DED maintains powder or wire deposition rates much higher than SLM and EBM. It leads directly to rapid prototyping of large-scale parts, resulting in a significant increase in cost-effectiveness and output. Moreover, DED is compatible with multi-axis machining tools, which facilitates the machining of trivial surface details and improves the integration of extensive post-processing. DED can also manufacture functionally graded materials (FGMs) in addition to various dense materials^16,17^. The hierarchical structure from SS316L to Inconel 718 to copper based on SS304L substrate was achieved by a DED system^18^. Stress distribution predicted by Finite Element Method (FEM) will be instructive when making material selection and design^19^. DED can also be used for surface coating, part maintenance, and the repair of defects such as cavities, cracks, and delamination^20–22^. For instance, Specifically, laser engineering net shaping (LENSTM) is a DED-based system for Inconel 718 internal defects repair^23^. Optical microscope observations reveal that the bonded component has good fusion and a smooth transition without void inclusions^20^.

Compressor discs, shafts, and turbine casings face fretting wear as one of their primary engineering challenges, which causes material loss, crack initiation, and a significant reduction in wear failure cycles^24,25^. Fretting wear is the multidisciplinary behavior of near-interface damage resulting from small oscillatory motions between two contact surfaces. With the assistance of DED, nickel-based alloys have the potential to be applied to seals, fasteners, marine instrument components, etc., whose shapes are difficult to process and prone to fretting wear^26^. Compare to wrought counterpart, DED-printed alloy tends to sacrifice surface properties and exhibit undesirable surface morphology^14,16,27^. This is a result of the partial alloy powder splash and balling effect, which causes a high roughness and poor wear resistance. For instance, the zirconia toughened alumina (ZTA) manufactured by DED exhibited poor flatness quality and roughness^28^. Consequently, the undesirable tribological properties severely restrict the prospective application fields^29^. To define and expand the field of alloy 718 application, a surface quality inspection and subsequent modification are required.

As an advanced post-processing technique, UNSM can enhance near-surface properties of a substance and enable a wide range of applications^30–32^. UNSM induces surface severe plastic deformation (SSPD) along with a gradient grain layer in the microstructure, which modifies the mechanical properties and surface roughness of material, thereby lifting its wear resistance^33^. The grain size and its distribution are the primary manifestations of microstructure, and UNSM is capable of refining coarse grains into nano-grains (as small as 50 nm) with a gradient shift in its distribution^34^. The grain size is finer closer to the surface, and it gradually increases in the direction of depth. There are quite a lot experimental results showing that UNSM technology plays an effective role in optimizing mechanical and tribological properties^35–37^. The ability to optimize surface quality by adjusting well-defined process parameters such as normal load, amplitude, frequency, and feed rate is one of the greatest benefits of UNSM technology^34^. The average incremental values for roughness and hardness brought by UNSM for a given set of process parameters are 58% and 27%, respectively^38^. Moreover, the COF and wear coefficient of Inconel 718 are reduced by 18.18% and 13.91%, respectively.

Although fretting wear simulation is acknowledged as a significant engineering issue, it is a relatively new area of computational research. Notably, FEM has become one of the most effective ways for analyzing the mechanical properties of various materials, thanks to the rapid evolution of computing power^39^. Archard's model and the dissipated energy model have been the most prevalent fretting wear calculation algorithms over the past two decades. On the basis of the Archard's model, a FEM-based standard numerical procedure for fretting wear process has been developed^24^. Fouvry and Sauger^40,41^ conducted additional research pertaining to the energy-based fretting wear calculation and its applications. In addition, the evolution of fretting wear parameters of this high-strength steel used in aeronautical engine transmission components was investigated.

In contrast to previous research, the fretting wear behavior of Inconel 718 manufactured by DED is inadequate. In addition, it is necessary to examine in depth how UNSM enhances the wear resistance of alloy 718. To investigate the effect of UNSM on wear performance, a finite element fretting wear model based on the energy dissipation model is developed in this work. In addition, the effect of each UNSM optimization parameter on fretting wear performance is investigated in order to gain a deeper understanding of the mechanism of UNSM in enhancing the wear resistance of materials.

## Experimental details

### Materials and fretting test rig

**Figure 1:**
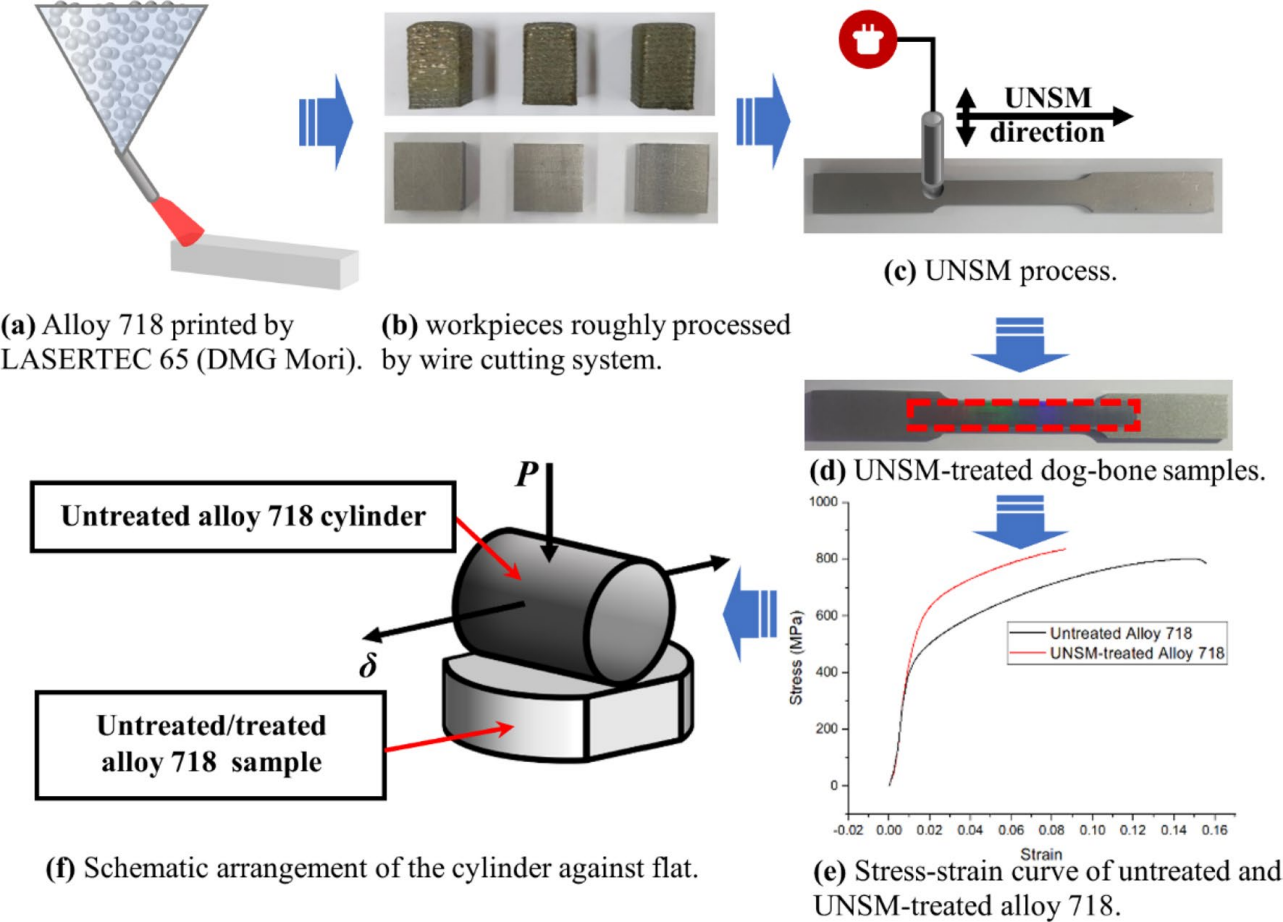
Main stages of alloy 718 samples preparing (**a**–**d**), arrangement for fretting wear test (**e**), and uniaxial tensile test result (**f**). Note: Fig. 1a, b, c, d, and e were created by Microsoft PowerPoint (Version 2110).

The primary sample preparation steps for analyzing the mechanical properties of alloy 718 manufactured by DED are depicted in Fig. 1. Initially, the samples are printed by LASERTEC 65 (DMG Mori) from Inconel 718 powders, whose principal composition is detailed in Table 1. Using a wire-cutting system, the alloy 718 samples are then formed into a dog-bone shape. The surface of the samples is then processed with UNSM, resulting in the formation of a hardening layer. Due to its extremely high hardness, tungsten carbide is chosen as the strike ball material in the UNSM process. The processing parameters are listed in Table 2.

**Table 1:** The chemical composition of the alloy 718.

Element [% weight]	Ni	Fe	Cr	Nb + Ta	Mo	Ti	Mn	Al
Alloy 718	Balance	18.0	18.0	4.98	2.99	1.02	0.12	0.02

**Table 2:** Parameters of UNSM treatment process.

Static load [N]	Frequency [Hz]	Interval [mm]	Amplitude [µm]	Ball dia. [mm] and material
50	20,000	0.03	30	2.38, tungsten carbide

The QMESYS (QM100T) universal tensile machine is used to conduct ASTM E8-standard uniaxial tensile tests on both UNSM-treated and untreated samples of alloy 718. Consequently, a comparison of the mechanical properties of alloy 718 before and after UNSM treatment is presented in Fig. 1e. The main mechanical properties of alloy 718 are elastic modulus, *E*, Poisson’s ratio, *ν*, yield stress, $\normalsize {\sigma }_{y}$, and ultimate tensile stress (UTS), as shown in Table 3. It is noteworthy that the alloy 718 produced by DED exhibited inferior mechanical properties. Nonetheless, the primary objective of this study is to investigate the impact of UNSM technology on fretting wear properties and consequently, the modification of tribological parameters will be emphasized.

**Table 3:** Mechanical properties of untreated and UNSM-treated alloy 718.

Samples	*E *[GPa]	*v*	${\sigma }_{y}$ [MPa]	UTS [MPa]
Untreated alloy 718	44.3	0.3	439	800
UNSM-treated alloy 718	45.5	0.3	570	835

**Figure 2:**
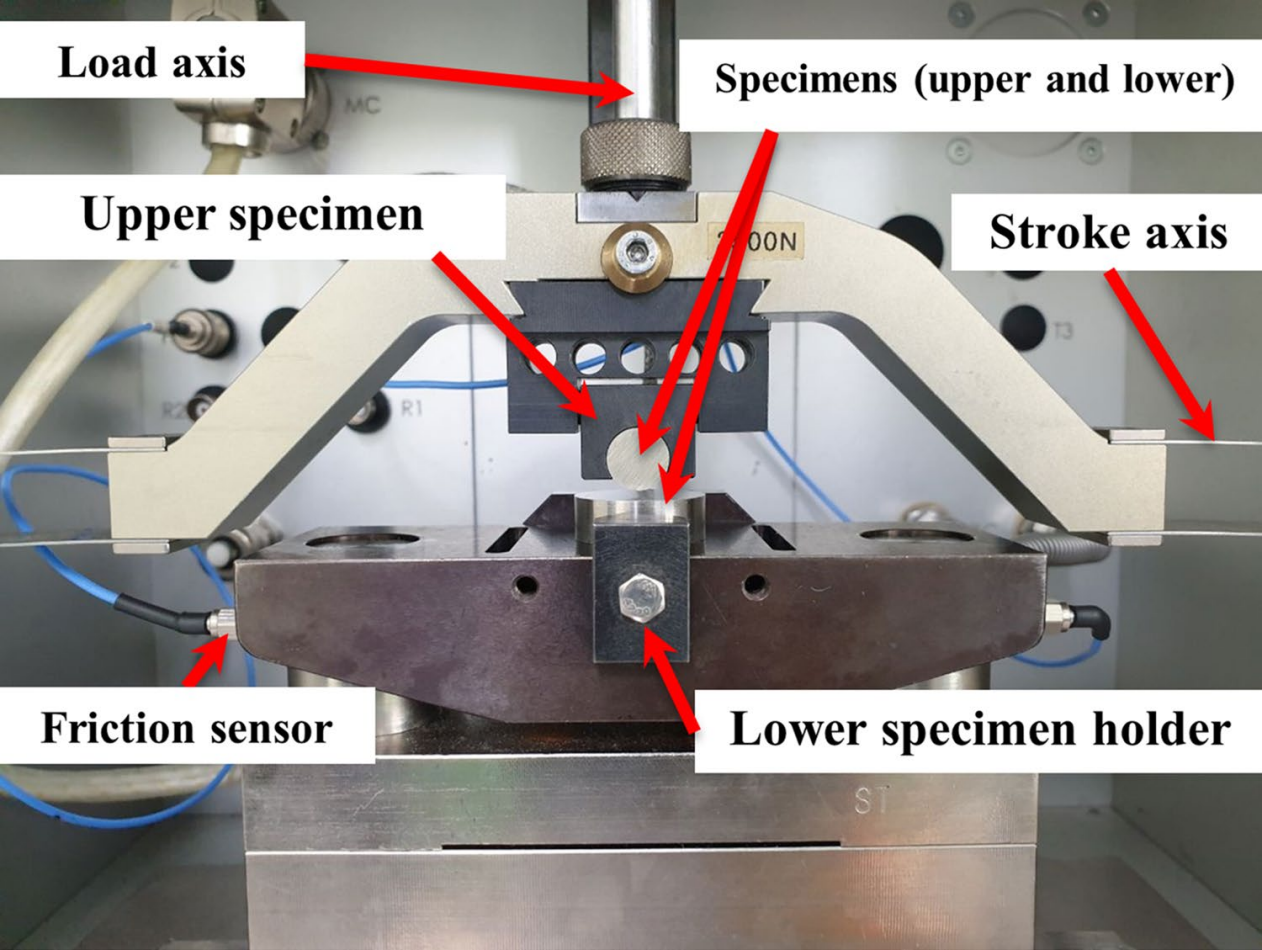
Diagrammatic view of the SRV-5 test system.

**Figure 3:**
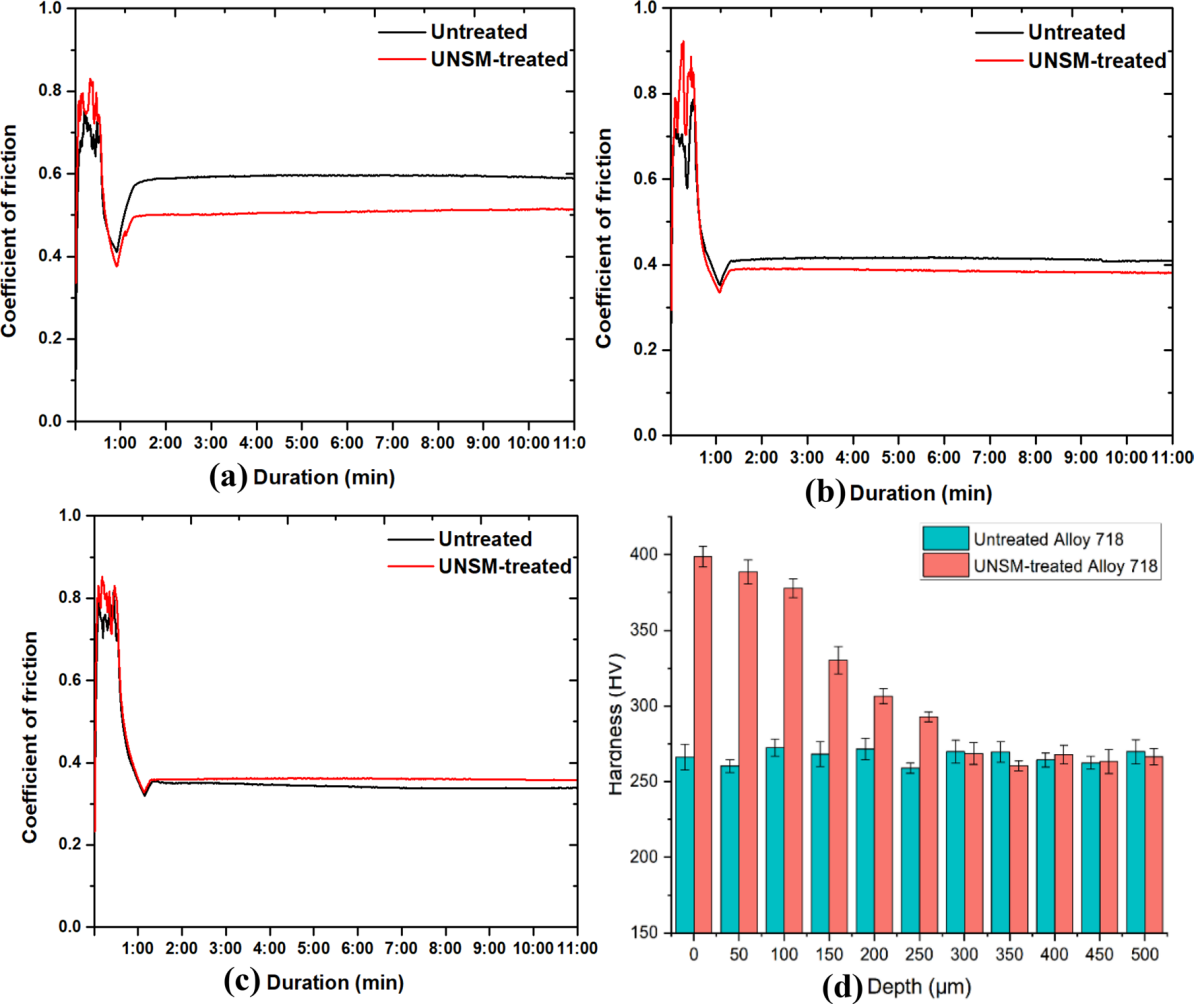
COF curves under different normal loads: 627 N (**a**), 880 N (**b**), and 1005 N (**c**); and hardness changes with depth (**d**) before and after UNSM treatment.

As shown in Fig. 1f, a cylinder-on-plane configuration is established to perform the fretting wear process. A series of fretting tests are carried out with the SRV-5 oscillation rig based on the cylinder/plane arrangement, which is shown in Fig. 2. The untreated/treated alloy 718 planes are clamped on the lower specimen holder, while the cylinder of untreated alloy 718 is constrained by the upper holder. To achieve the fretting behavior, a small amplitude oscillatory displacement, *δ*, is applied along the radial direction of the cylinder horizontally by stroke axis. Additionally, the load axis gives a constant normal load, *P*, on the upper cylinder to reach the purpose of contact. During the fretting process, the friction sensor measures the applied displacement and the tangential traction force, which will be converted into the curve of COF. For UNSM-treated specimen, the changes in microstructure can be evaluated by the gradient hardness. In this study, a Micro-Vickers hardness tester (Mitutoyo MVK-E3, Tokyo, Japan) is utilized to determine the hardness along the depth, as shown in Fig. 3. Notably, the hardness measurements for each depth were repeated four times. It can be observed that, when the depth reaches 300 µm, the hardness of the UNSM-treated sample tends to be stabilized. Furthermore, the hardness value is not significantly different from that of the untreated sample, indicating that UNSM has little effect on the microstructure at depths greater than 300 µm. Given the processing parameters used for UNSM in this study, the maximum depth affected by UNSM is therefore 300 µm.

### Tribological properties

In addition to changes in mechanical properties, the UNSM treatment influences the tribological parameters such as COF and wear coefficient of the alloy 718 samples. The effectiveness of the UNSM technique on the wear resistance of the alloy 718 under various loading conditions is studied. Considering the severe working conditions, the normal load *P* is set to 627 N, 880 N, and 1005 N, respectively. Due to the fact that the magnitude of the stress state of alloy 718 during the fretting wear process will exceed its yield stress, thereby plastic deformation will occur. At this point, with the introduction of the hardening model, which will be described in the finite element simulation section, the plastic behavior can be incorporated into this study. The half sliding distance *δ* = 120 μm, and the fretting frequency is set to 30 Hz, so it takes approximately 11 min to complete 20,000 cycles. COF is defined as the ratio of measured friction force amplitude during one cycle to applied normal load *P* during the fretting wear process:

1 $$\mu =\frac{{f}_{max}}{P}$$

Therefore, the evolution of COF versus time under various normal loads are plotted in Fig. 3a–c. There are steep peaks and valleys that occurred during running-in stage, i.e., up to about 1.5 min from beginning of test. The curve of COF then stabilizes at a constant value during the steady state stage of fretting wear. After the complement of fretting test, the wear volume of surface is measured with a profilometer (Mitutoyo SJ-210, Kanagawa, Japan) and then will be validated with the finite element results. In addition, according to the fretting wear algorithm used in finite element model, i.e., the dissipated energy model, the wear loss measured by the experiment will be calculated as the energy-based wear coefficient in the next section. Consequently, the wear coefficient will be transferred to the finite element model for subsequent fretting wear simulations.

### Finite element simulation

#### Basic contact model

**Figure 4:**
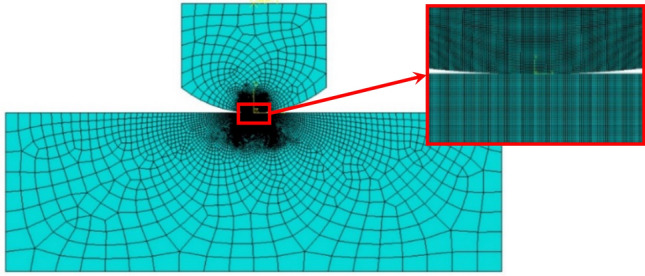
Schematics of contact model in FEM (Abaqus v6.19). Note: Fig. 4 was generated by ABAQUS (v6.19).

As depicted in Fig. 4, a finite element model of the cylinder/plane configuration was constructed in a commercial software of ABAQUS to correspond to the experimental setup^42^. Considered as a problem of plane strain, a two-dimensional finite element model is constructed. Four-node plane strain element (CPE4) are specified in the cylinder and plane parts. Table 4 displays the dimensions of finite element model, where the radius and axial length of cylinder are 5.5 mm and 15 mm, respectively. The length and thickness of specimen are 24 and 8 mm, respectively. The contact regions of the cylinder and specimen are divided into two parts, resulting in a finer mesh to capture the high stress gradient occured. In addition, rectangular elements are meshed in the contact regions, which facilitates the output data in the post-processing step. Multi-point constraints (MPCs) are imposed on the top surface of the pad to ensure that the set of nodes have equal degrees of freedom. Hence, implausible deformation and rotation caused by concentrated loads will be avoided. Both the two sides and bottom of specimen are constrained along the *x* and *y* axes. For the materials, untreated alloy 718 (as an upper side cylindrical intender) and untreated/treated alloy 718 (as a lower side flat sample) are utilized in this study. The mechanical properties are consistent with the experimental data, as shown in Table 3.

**Table 4:** Dimensions of the 2D contact model.

Model	${R}_{c}$[mm]	${L}_{c}$[mm]	${L}_{s}\times T$[mm^2^]
Cylinder on plane	5.5	15	24 × 8

#### Wear profile update in FEM

To calculate the wear loss, various wear laws are studied in the references^24,40,41^. The most widely used contact-mechanics-based formula for wear calculating are Archard’s model and dissipated energy model. For Archard’s wear model, the specific wear rate can be expressed as:

2 $$k = \frac{V}{P \cdot s}$$

where $F$ is the load, $V$ is the wear loss volume, and $s$ is the tangential sliding distance. The local wear depth within $\Delta N$ jump cycles is obtained by McColl et al. based on a modified Archard’s equation:

3 $$\Delta y\left(x\right)={k}_{A}\cdot p\left(x\right)\cdot 4\delta \left(x\right)\cdot \Delta N$$

where ${k}_{A}$ is the Archard’s wear coefficient, which is assumed to be a constant along the contact surface. In addition, $p(x)$ and $\delta (x)$ represent the local contact pressure and local relative sliding amplitude, respectively.

However, in many numerical simulation results, the dissipated energy model tends to be in better agreement with experimental results. Particularly, wear prediction using Archard's model is less accurate under conditions of high contact pressure. Thus, the energy wear model is selected to integrate the wear calculation with stress into FEM in this paper. Paulin propose that the wear volume is proportional to a portion of the frictional work (dissipated energy)^43^. It will induce a structural transformation, allowing FEM to capture the plastic behavior within wear cycles. Experimental measurements indicate that the total wear volume is proportional to the energy dissipated as follows:

4 $$V={k}_{E}{\sum }_{i}^{N}{E}_{{d}_{i}}$$

where ${\sum }_{i}^{N}{E}_{{d}_{i}}$ represents the accumulated dissipated energy and ${k}_{E}$ is the energy wear coefficient corresponding to this specific interface displacement amplitude, $\delta$. Based on Coulomb’s law, the energy-based wear coefficient can be written as:

5 $${k}_{E}=V/{\sum }_{i}^{N}4{\mu }_{i}{P}_{i}{\delta }_{i}$$

where ${\mu }_{i}$ is the coefficient of friction and ${P}_{i}$ is the normal load in fretting cycle of ${i}$th. In addition, using FEM, the variability of COF has a minimal effect on the high-cycle wear loss in the gross sliding regime^44^. In light of this, COF can be viewed as a weighted average of the steady-state portion of the testing curve, which will then be fed into the FEM for prediction. Under a constant normal load *P* and a sliding displacement amplitude $\delta$ = 120 µm, the entire procedure lasts 20,000 cycles. In this instance, the energy-based wear coefficient, ${k}_{E}$, can be expressed as follows:

6 $${k}_{E}=\frac{V}{{9600}\cdot \mu \cdot P}$$

Consequently, the energy wear coefficient for each normal load *P* is calculated using Eq. (6) and listed in Table 6. In addition, the incremental wear depth after $\Delta N$ jump cycles can be computed as follows:

7 $$\Delta {y}_{i}(x)={k}_{E,local}\cdot {q}_{i}(x)\cdot \Delta N\cdot d{\delta }_{i}(x)$$

where ${k}_{E,local}$ is local energy wear coefficient, ${q}_{i}(x)$ is local shear stress, and $d{\delta }_{i}(x)$ is the relative sliding distance of ${i}$th fretting cycles at $x$-coordinate. Notably, the local energy wear can be considered equal to $\normalsize {k}_{E}$, i.e., $\normalsize {k}_{E,local}={k}_{E}$.

#### Wear algorithm in ABAQUS

Since the wear process occurs primarily in the specimen's contact area, the Arbitrary Lagrange-Eulerian (ALE) adaptive meshing technique can be used to update the surface contour. In the contact area, the ALE domain is uniquely designated, and its upper surface is designated as the ALE node. UMESHMOTION is presented as a calculator that computes the incremental local wear depth using the dissipated energy method. The current wear depth at ${i}$th fretting cycles can be expressed as:

8 $${y}_{i}\left(x\right)={y}_{i-1}(x)-\Delta {y}_{i}(x)$$

After obtaining updated $y$-coordinates of the top surface of the specimen, the coordinate of the nodes will be fed into the ALE technique. Thus, the surface nodes in the contact zone of the specimen will be adjusted to the new positions and a new mesh will be generated in the contact area. In this manner, the evolution of the worn surface will be simulated step by step alongside the fretting cycles.

#### Loading history and boundary conditions

**Figure 5:**
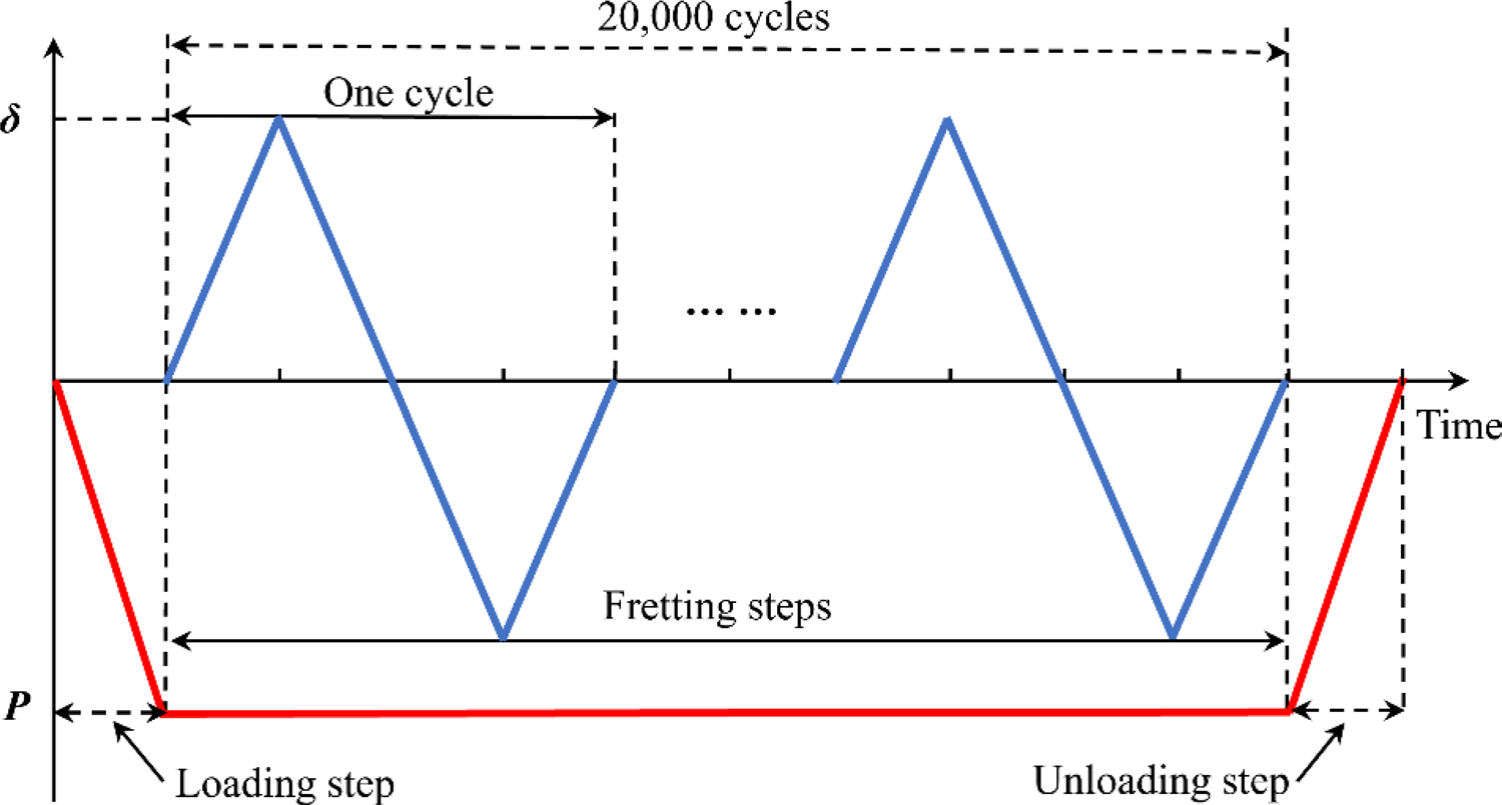
Loading and displacement history in fretting wear model.

Compared to other forms of damage, fretting wear has a dominant role in gross sliding condition. The loading history is shown in Fig. 5, where a normal load $P$ is applied in the centre of the upper surface of the pad within loading step. To study the fretting wear behavior under severe conditions, the sliding state between contact surfaces in this paper is set as global slip. The movement of the cylinder with a tangential amplitude of *δ* = 120 μm is defined as a boundary condition in variant displacement in sliding steps. After 20,000 fretting cycles, the normal load $P$ is withdrawn in the unloading step.

#### Plasticity

Since critical components are often exposed to harsh operating conditions, not only due to the wear performance of alloy 718 at large fretting distances, but also due to high normal loads. The cylindrical intender is subjected to three relatively high loads in the range of 627–1005 N, as previously described. Moreover, the existence of plastic strain induced by large stress state has an important influence on the surface stress state distribution and wear resistance. It shows in the energy-based wear model of Eq. (7) that the local stress ${q}_{i}(x)$ is important for incremental wear depth calculation and worn surface update. Therefore, it is necessary to capture the plastic behavior of alloy 718 samples under high loading conditions.

**Figure 6:**
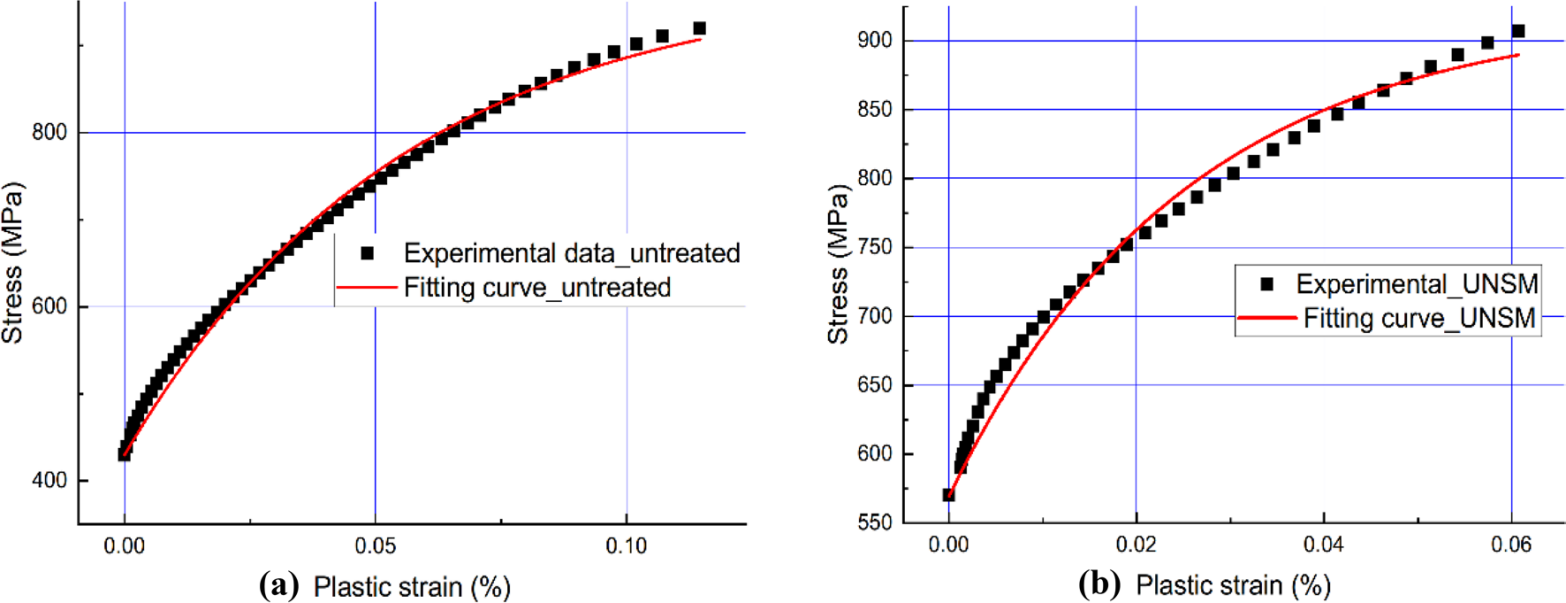
Comparison between experimental data and fitting curves: as-printed (**a**), and UNSM-treated (**b**) alloy 718.

In this case, the linear isotropic hardening law is employed in the constitutive model to incorporate plasticity. It is associated with an accumulated dislocation structure that expands the yield surface of the material under plastic deformation. The tendency for yield stress to increase linearly with increasing plastic strain was found in the isotropic hardening model^45^. In the case of von Mises loading surface, the evolution of the yield surface can be expressed as a function related to the equivalent plastic strain ${\varepsilon }_{pl}$:

9 $${\sigma }^{0}={\left.\sigma \right|}_{0}+Q(1-{e}^{-b{\varepsilon }_{pl}})$$

where ${\left.\sigma \right|}_{0}$ is the yield stress at zero plastic strain, $Q$ and $b$ are material parameters. In detail, $Q$ is the maximum change in the size of the yield surface, and it also indicates the hardening saturation property. Material parameter $b$ defines the rate, at which the yield surface size changes with the evolution of the plastic strain. In this study, each hardening parameter for the alloy 718 sample is determined by curve fitting based on Eq. (9). According to the experimental data of uniaxial tensile test, the curve of fitting equation is shown in Fig. 6, and the hardening parameters are listed in Table 5.

**Table 5:** Main hardening parameters of Johnson-cook model for alloy 718.

Sample	${\left.\sigma \right|}_{0}$ [MPa]	$Q$ [MPa]	$b$
As printed	439	547	18
UNSM-treated	570	351	40

## Simulation procedure

**Figure 7:**
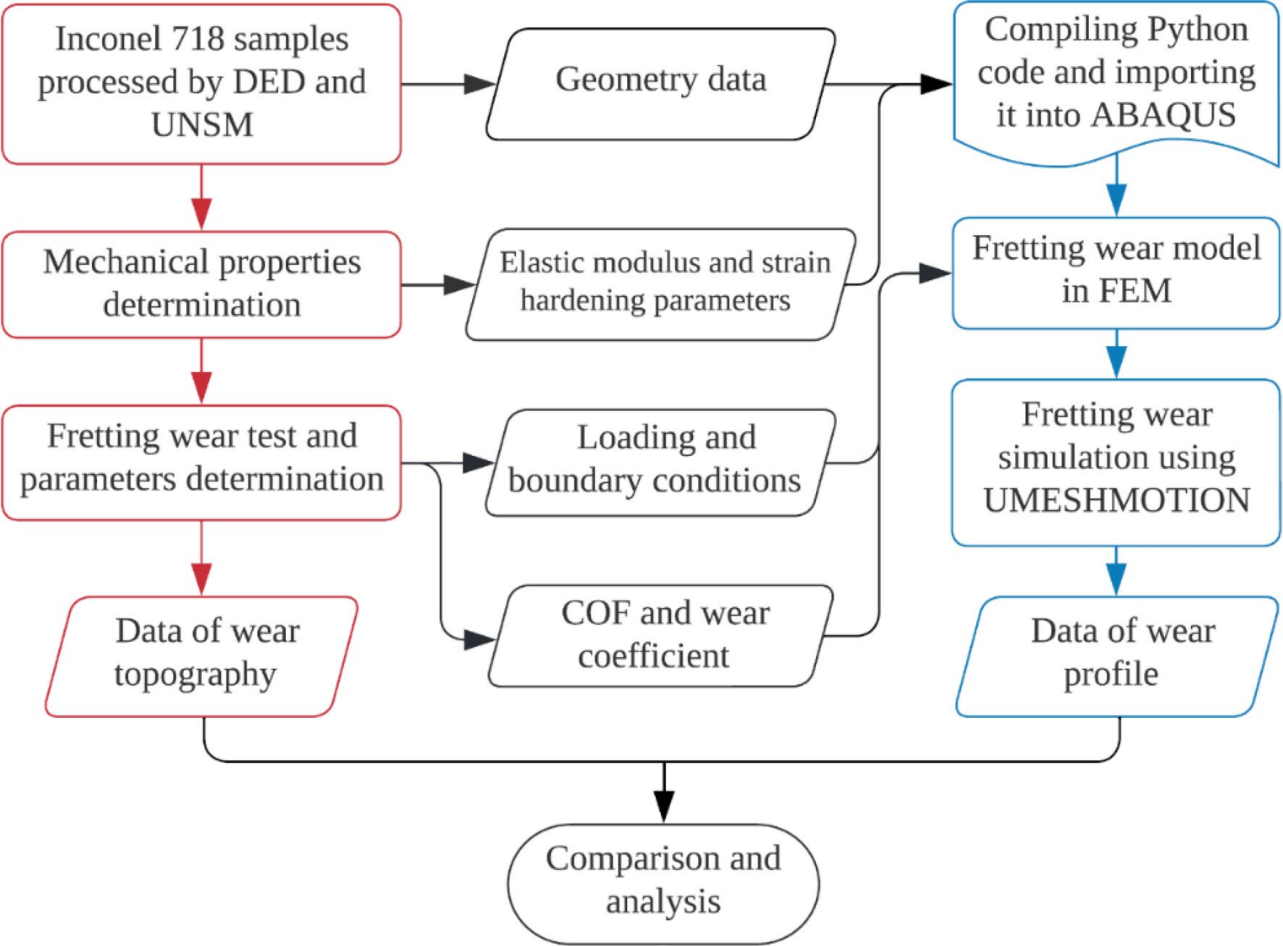
Flow chart of the experiment and finite element simulation.

According to the experimental test, alloy 718 sample after UNSM treatment exhibits higher mechanical and tribological properties. The main factors modified by UNSM include Young’s modulus $E$, hardening parameters, COF, and wear coefficient. To investigate the effect of UNSM on wear resistance using FEM, the first step is to extract related resulting factors from experimental test to FE simulation. As shown in Fig. 7, these material parameters will be transferred to Python code and user’s subroutine UMESHMOTION with geometry data to generate fretting wear model in FEM. Notably, due to the algorithms used for wear depth update (dissipated energy model), fretting wear profile changes are primarily determined by the COF and wear coefficient with only a minor impact on the contact pressure distribution. Consequently, the ignored modeling of gradient structure and residual stress has a negligible effect on the accuracy of numerical simulation.

Benefiting from the flexibility of FEM, not only can the overall impact of UNSM on the 718 alloy be considered, but also the effect of each parameter modified by UNSM. As shown in Table 6, 18 cases are designed to consider the effect of UNSM overall, UNSM-optimized COF, and UNSM-optimized wear coefficient on wear properties, respectively.

**Table 6:** Effect of UNSM on surface properties of alloy 718 and parameter examination cases.

Parameter examination	Cases	*P* [N]	Samples	$E$ [GPa]	COF	${K}_{E}$ [MPa^−1^]
Overall	1	627	as printed	44.3	0.59	9.96 × 10^−9^
	2		UNSM-treated	45.5	0.52	7.73 × 10^−9^
	3	880	as printed	44.3	0.43	11.54 × 10^−9^
	4		UNSM-treated	45.5	0.41	9.94 × 10^−9^
	5	1005	as printed	44.3	0.36	12.44 × 10^−9^
	6		UNSM-treated	45.5	0.38	11.19 × 10^−9^
COF	1	627	as printed	44.3	0.59	9.96 × 10^−9^
	1.1		UNSM-treated	44.3	0.52	9.96 × 10^−9^
	3	880	as printed	44.3	0.43	11.54 × 10^−9^
	3.1		UNSM-treated	44.3	0.41	11.54 × 10^−9^
	5	1005	as printed	44.3	0.36	12.44 × 10^−9^
	5.1		UNSM-treated	44.3	0.38	12.44 × 10^−9^
${K}_{E}$	1	627	as printed	44.3	0.59	9.96 × 10^−9^
	1.2		UNSM-treated	44.3	0.59	7.73 × 10^−9^
	3	880	as printed	44.3	0.43	11.54 × 10^−9^
	3.2		UNSM-treated	44.3	0.43	9.94 × 10^−9^
	5	1005	as printed	44.3	0.36	12.44 × 10^−9^
	5.2		UNSM-treated	44.3	0.36	11.19 × 10^−9^

The overall effect of UNSM under different normal loads is integrated into Cases 2, 4 and 6 and then will be validated with the finite element model. Cases 1.1, 3.1 and 5.1 are devoted to studying the effect of UNSM-optimized COF on wear performance. Similarly, Cases 1.2, 3.2 and 5.2 were used to study the effect of UNSM-optimized wear coefficient on the wear characteristics. The control variable method is used here, for example, when the wear coefficient is to be checked, other parameters such as the constitutive model and COF will be set to the same parameters in both models. To test the effect of each parameter, the untreated 718 alloy samples are marked as Cases 1, 3 and 5, and then used as a control group to compare with other cases.

## Results and discussion

### Validation of the finite element model

Hertz^46^ developed a theory for estimating the pressure distribution and contact area between two contact surfaces under applied normal loads. Consequently, the Hertzian contact equations can be used to validate the accuracy of the contact behavior in finite element model. The primary aspect of validation is the contact pressure distribution, which consists of contact peak pressure and contact width. Regarding the Hertzian contact theory, the effective radius, $\overline{R}$, for cylinder-on-flat configuration can be expressed as follows:

10 $$\overline{R}={R}_{c}$$

where ${R}_{c}$ is the radius of the cylinder. The effective elastic modulus $\overline{E}$ is define below using Young’s modulus ${E}_{c}$, ${E}_{s}$ and Poisson’s ratio ${\nu }_{c}$, ${\nu }_{s}$ of cylinder and specimen, respectively:

11 $$\frac{1}{\overline{E}}=\frac{1-{\nu }_{s}^{2}}{{E}_{s}}+\frac{1-{\nu }_{c}^{2}}{{E}_{c}}$$

12 $$w=\sqrt{\frac{4P\overline{R}}{\pi \overline{E}L}}$$

Therefore, the contact half width and pressure distribution are given as:

13 $$p(x)={p}_{0}\sqrt{1-{\left(\frac{x}{w}\right)}^{2}}\mathrm{where }\ {p}_{0}=\sqrt{\frac{P\overline{E}}{\pi \overline{R}L}}$$

where $L$ is the line contact length and ${p}_{0}$ is the maximum Hertzian contact pressure.

**Figure 8:**
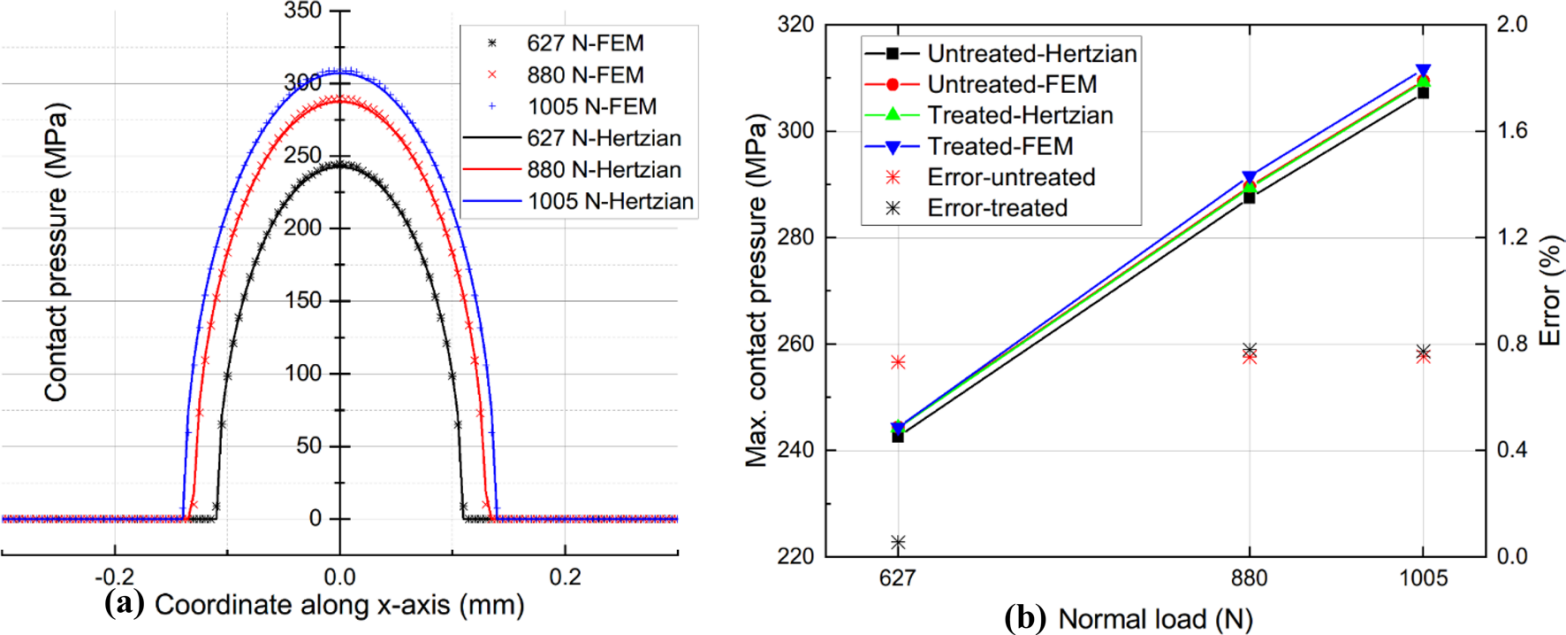
Comparison of contact pressure distribution (**a**), and maximum contact pressure values (**b**) between Hertzian and FE solutions under various normal loads.

Take an example for untreated alloy 718, the contact pressure distribution from FEM and the Hertzian solution are compared in Fig. 8 when the normal load is 627 N, 880 N, and 1005 N, respectively. It demonstrates that the contact pressure distribution derived from the FE model agrees well with the analytical solution. Specifically, the FE models for both untreated and UNSM-treated samples exhibit maximum contact pressure errors of less than 1%. It indicates that the FE model in this study is properly implemented with loading and boundary conditions, mesh size, and mesh geometry.

### Effect of UNSM on wear performance

The global impact of UNSM on wear performance is analyzed, and the results are compared to experimental data to validate the accuracy of the finite element model. It is challenging to specify which parameters play a significant role and what role each individual plays in the fretting wear process. Consequently, it is necessary to consider not only the overall effect of UNSM, but also the impact of each UNSM-modified parameter on the wear performance. The experimentally measured mechanical properties in Table 3 reveal that the elastic modulus *E* optimized by UNSM is marginally greater than that of the untreated, i.e., 45.5 and 44.3GPa, respectively. The contact pressure amplitude difference between untreated and UNSM-treated samples is minimal. Even if the normal load *P* reaches 1005 N, the difference between Hertzian theory and FEM calculations is only about 1%. Thus, it is unnecessary to investigate only the effect of the UNSM-optimized elastic modulus *E* on wear performance.

### Overall

**Figure 9:**
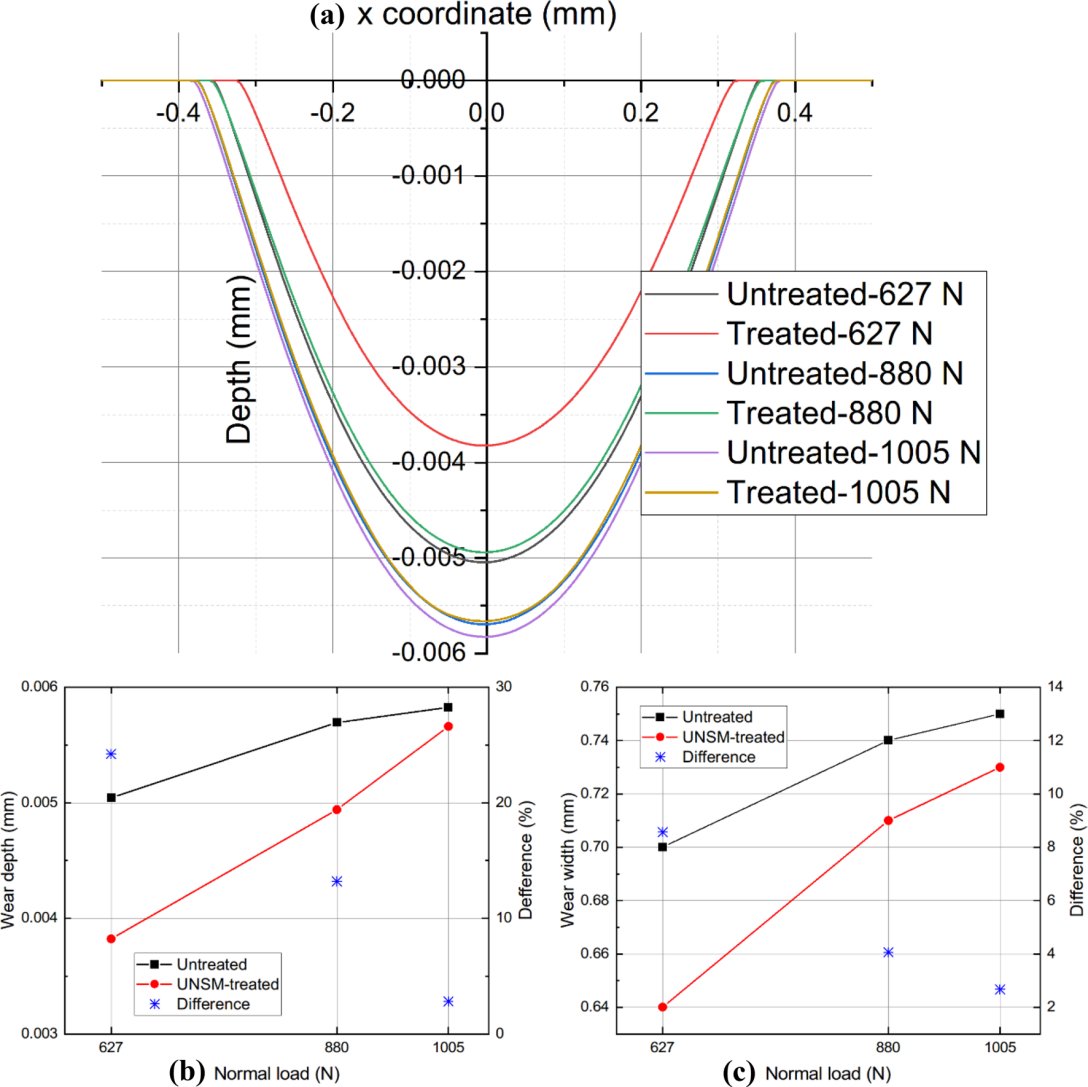
Comparison of wear profiles (**a**), wear depth (**b**), and wear width (**c**) for untreated and UNSM-treated alloy 718 under various normal loads.

**Figure 10:**
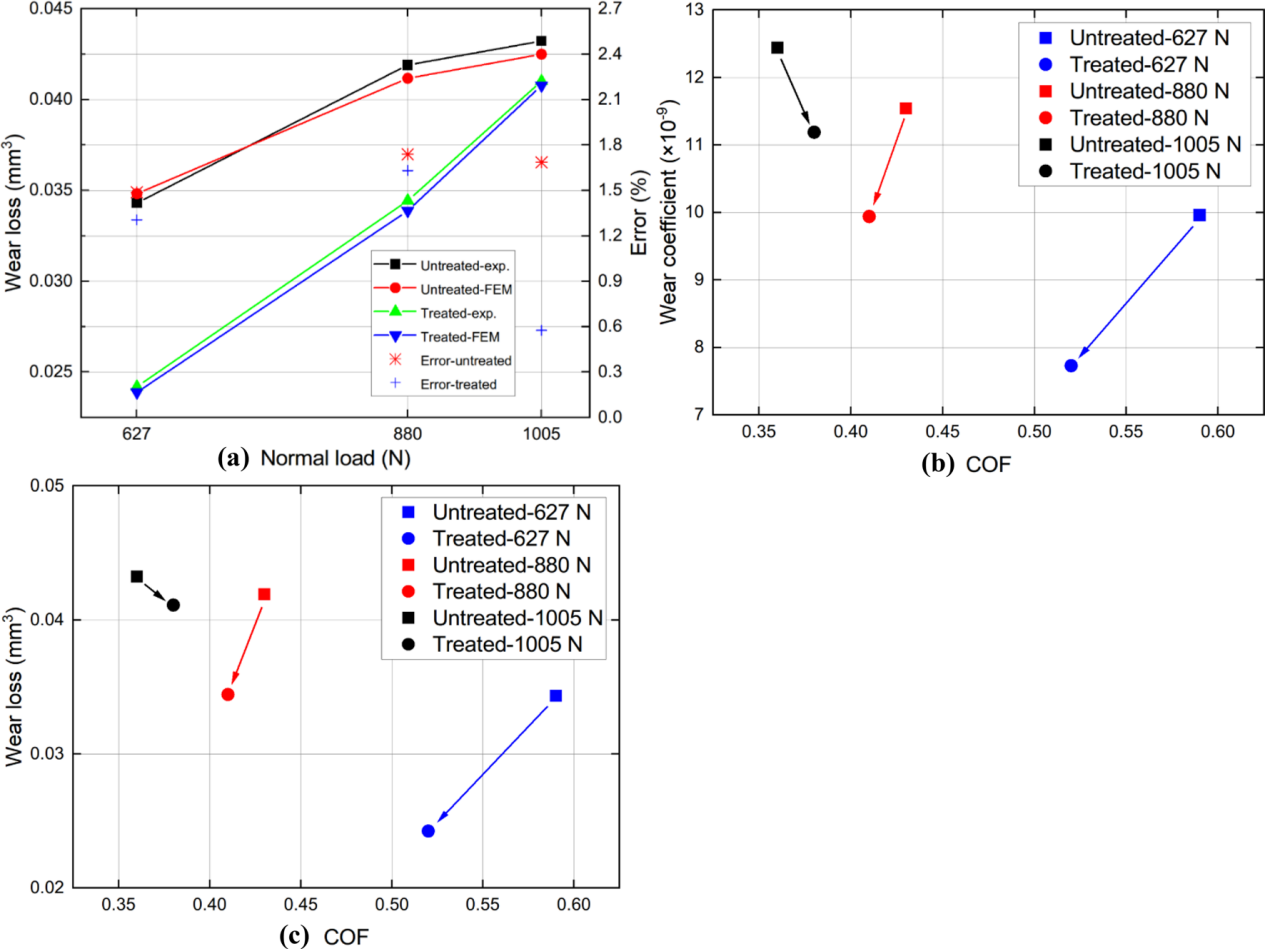
Comparison of wear loss between experimental and FEM solutions (**a**), COF and wear coefficient (**b**), and COF and wear loss (**c**) for untreated and UNSM-treated alloy 718 under various normal loads.

Considering the overall effect of UNSM, the wear scar produced by FEM between UNSM-treated and as-printed alloy 718 are plotted in Fig. 9. It was observed that with the increase of normal load *P*, the wear scar became larger and deeper. Owing to UNSM, when *P* = 627 N, the wear depth is reduced from 0.0050 mm to 0.0038 mm after 20,000 cycles, a reduction of 24.22%. And when *P* reaches 1005 N, the maximum wear depth reaches about 0.0058 mm, which means that the specimen is in a relative unfavorable working condition. In addition, the mitigation degree of UNSM on wear depth dropped to 2.82%. Similarly, the relief degree of UNSM on wear width is decreased from 8.57% to 2.67% also. The wear volume can be calculated by integrating the area above the curve and then be compared with the experimental data. It exhibits an unprecedented conformity, and the maximum error between FEM and experimental result is less than 2%, as shown in Fig. 10a. It becomes evident that the fretting wear model built in this study can be used to predict the volume of material loss caused by the fretting wear process accurately. Notably, Figs. 10b and 10c depict the relationship between COF and wear coefficient and COF and wear loss, respectively.

**Figure 11:**
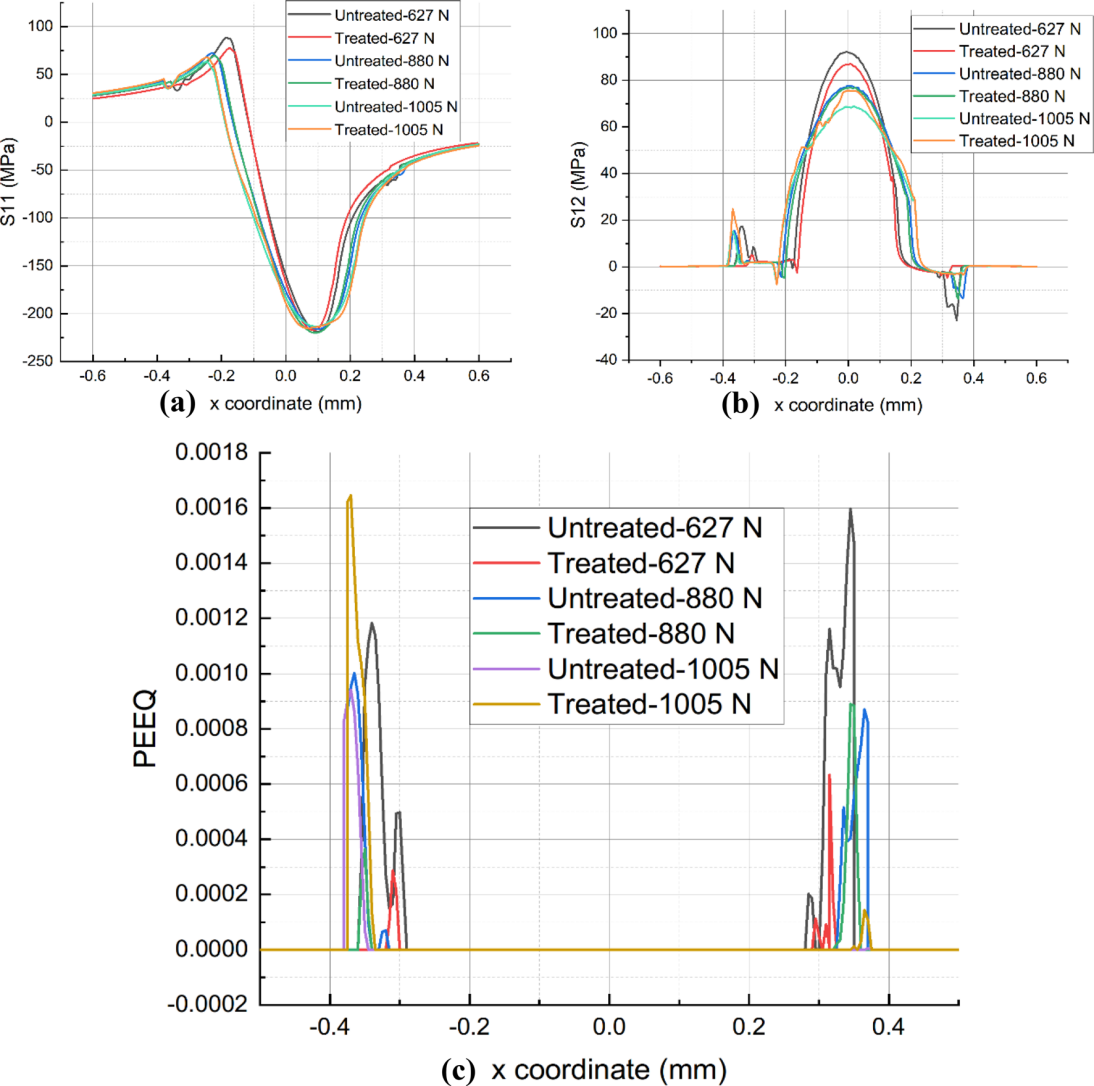
Comparison of S11 (**a**), S12 (**b**), and PEEQ (**c**) for as-printed and UNSM-treated alloy 718 under various normal loads.

By examining the stress and strain distributions on the contact surfaces, it is possible to comprehend the impact of UNSM on wear performance. Figures 11a and 11b depict the stress distributions S11 and S12 for the untreated and UNSM-treated samples at the end of the final fretting cycle. S11 represents the normal stress along the $x$-coordinate and its amplitude occurs at the contact edge between the sample of alloy 718 and the cylinder. Regardless of the value of *P*, the magnitude of S11 at leading edge remains unchanged, the difference is within 2%. For S11 at the trailing edge when *P* is increased from 627 to 880 N, the UNSM-treated samples are 12.43% and 4.40% less than the untreated samples. However, when *P* reaches 1005 N, it increases by 7.30%. In addition, the position of the S11 amplitude moves outward, which is one of the main reasons for the widening of the wear width. Moreover, it is observed that the shear stress S12 of UNSM-treated one along the contact surface is reduced when *P* = 627 N and 880 N, whereas it increases instead when *P* = 1005 N. It is unclear whether this is primarily the result of the UNSM-modified COF or the wear coefficient.

Due to the high normal load *P*, the equivalent plastic strain (PEEQ) appears after the unloading step, as shown in Fig. 11c. PEEQ is a scaler variable of all the components of equivalent plastic strain at one point in the finite element model. In this work, PEEQ is used to equivalently represent the degree of plastic deformation produced in the specimen induced by fretting wear process. It is observed that PEEQ is mainly canyon-likely distributed near the edge of the wear scar, while it is zero in the middle area. This is due to the high contact pressure that is generated in the middle region under normal load *P*. In addition, the surface is updated by the Eqs. (7) and (8) based on normal load *P*, and there is a substantial loss of material in the central region. In addition, the surface is updated by the above-mentioned equation, which includes the term of the normal load *P*, so the closer to the central region, the greater the likelihood of a material loss is. Thereby, the plastic region in the middle surface area is removed after 20,000 fretting cycles, leaving only the elastic region. Specifically, UNSM-treated sample exhibits a lower amplitude of PEEQ compared with its untreated one when *P* = 627 N and 880 N. However, an exception exists when *P* = 1005 N. It is unclear how the parameters, specifically the UNSM-optimized COF and wear coefficient, led to this result. Therefore, the impact of each parameter will be evaluated to gain a deeper understanding of its intrinsic mechanism.

### Coefficient of friction

After a comprehensive analysis, the fretting wear model can be applied to investigate the influence mechanism of each parameter on the wear performance. The COF is necessary for the energy wear model to accurately predict the wear scar. When using variable COF, the wear scar differences between samples are found to be relatively small, especially when the fretting cycle reaches high values. In this study, the untreated COF and UNSM-optimized COF are evaluated under various loading conditions, and as shown in Table 6, only the constantly averaged COFs are adopted in this finite element model. Regardless of whether the samples were treated with UNSM or not, the value of COF decreases as *P* increases from 627 N to 1005 N. Moreover, because UNSM flattens the surface topology, the COF of UNSM-treated samples is lower than that of untreated samples for the majority of load cases. When *P* reaches 1005 N, however, the UNSM-modified COF value increases by 5.6% compared to its as-printed value.

**Figure 12:**
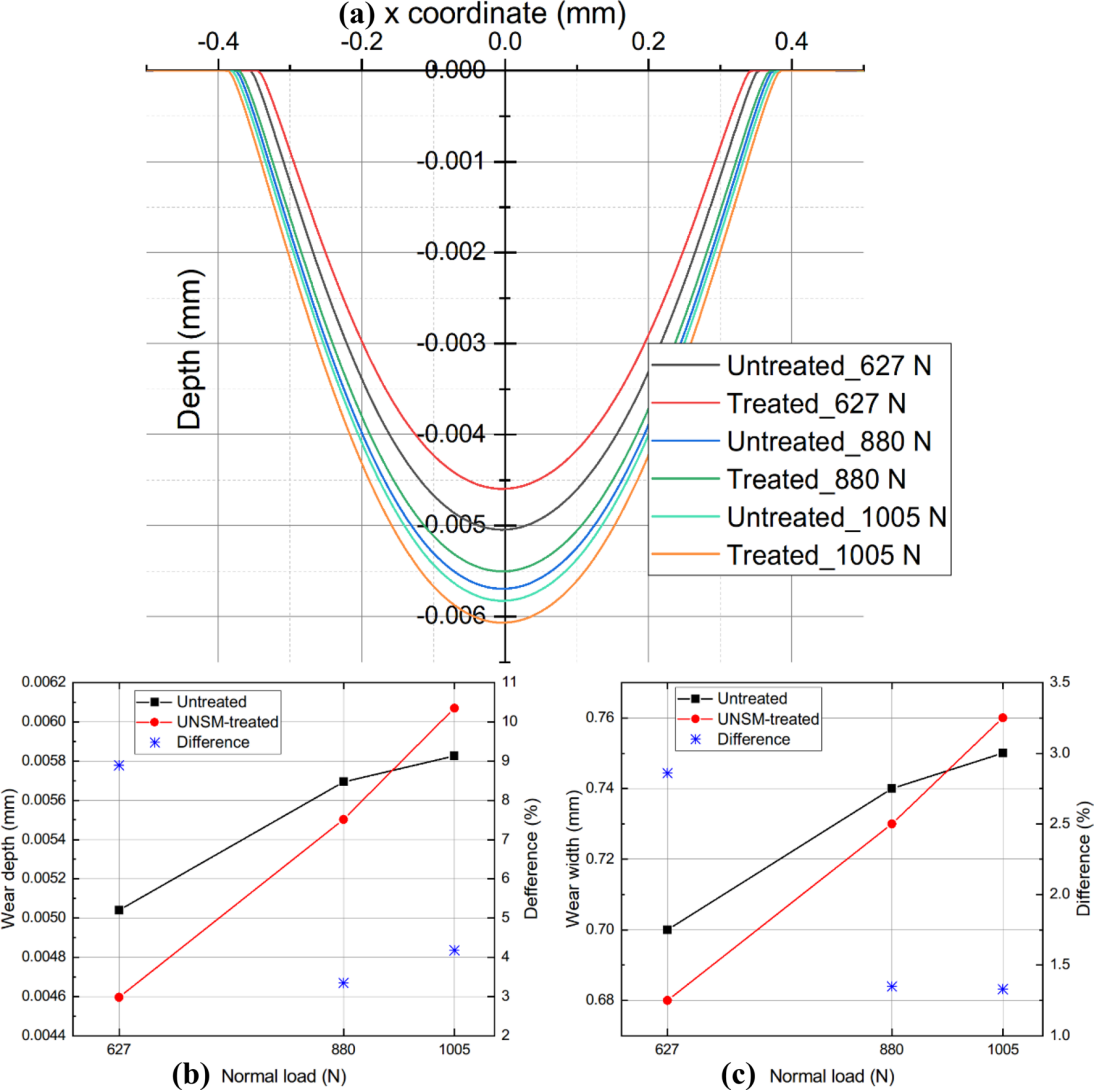
Comparison of wear profiles (**a**), wear depth (**b**), and wear width (**c**) for untreated and UNSM-treated alloy 718 with modified COF under various normal loads.

The objective of Cases 1.1, 3.1, and 5.1 is to observe the impact of UNSM-optimized COF on wear performance, while the untreated serves as the control case. Figure 12 depicts the simulation results examining the effect of the UNSM-modified COF on the wear scar and the comparative details. With increasing normal load $\it P$, the improvement in wear resistance brought about by the UNSM-modified COF gradually diminished. When *P* = 627 N, UNSM-modified COF has an exceptional 8.9% effect on wear resistance. Particularly, when *P* = 1005 N, it has a lower wear resistance than untreated material.

**Figure 13:**
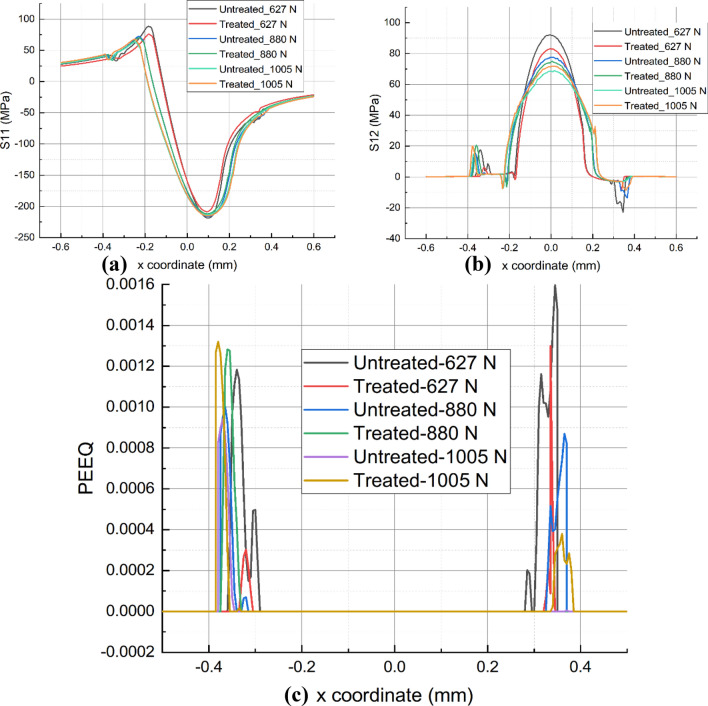
Comparison of S11 (**a**), S12 (**b**), and PEEQ (**c**) for as-printed and UNSM-treated alloy 718 with modified COF under various normal loads.

The effect of UNSM-modified COF on wear characteristics can also be observed from the stress distribution along the contact surface, as depicted in Figs. 13a and 13b. The amplitude of the *x*-direction normal stress, S11, is concentrated at the contact surface's edge. The degree of mitigation caused by the UNSM-modified COF is primarily concentrated at the trailing edge of the contact, particularly when *P* = 627 N, and the degree of relief reached 10%. With the addition of *P*, the improvement fells to approximately 2%, which is insignificant. Notably, when *P* = 1005 N, the S11 amplitude of UNSM-treated COF is marginally greater than that of untreated COF. This is primarily due to the abnormal increase in COF following UNSM therapy. Likewise, the magnitude of the shear stress S12 with the UNSM-modified COF is lower than that of the original sample. Moreover, under the same normal load, such as when *P* = 1005 N or 880 N, the UNSM-modified COF resulted in a smaller S12 magnitude. This is primarily due to the significant reduction of COF by the UNSM technique. In addition, the PEEQ distributed on the contact surface after the unloading step can be used to determine the effect of the optimized COF on the wear resistance, as depicted in Fig. 13c. The PEEQ is distributed like a canyon along the wear profile, with no value in the middle region. This is mainly due to the high contact pressure produced during the wear process, which causes the material in the intermediate surface region to be removed after a large number of fretting cycles. After unloading step, only the elastic region remains. Under the same normal load *P*, the UNSM-modified COF results in a weaker PEEQ distribution than the untreated one. It can be explained by the fact that a lower COF leads to a lower stress distribution, thereby reducing the accumulation of plastic strain. In turn, a lower COF results in a narrower wear width and the peak remaining closer to the interior. However, when *P* = 1005 N, the magnitude of PEEQ increases primarily due to the higher stress state caused by the higher COF.

### Wear coefficient

**Figure 14:**
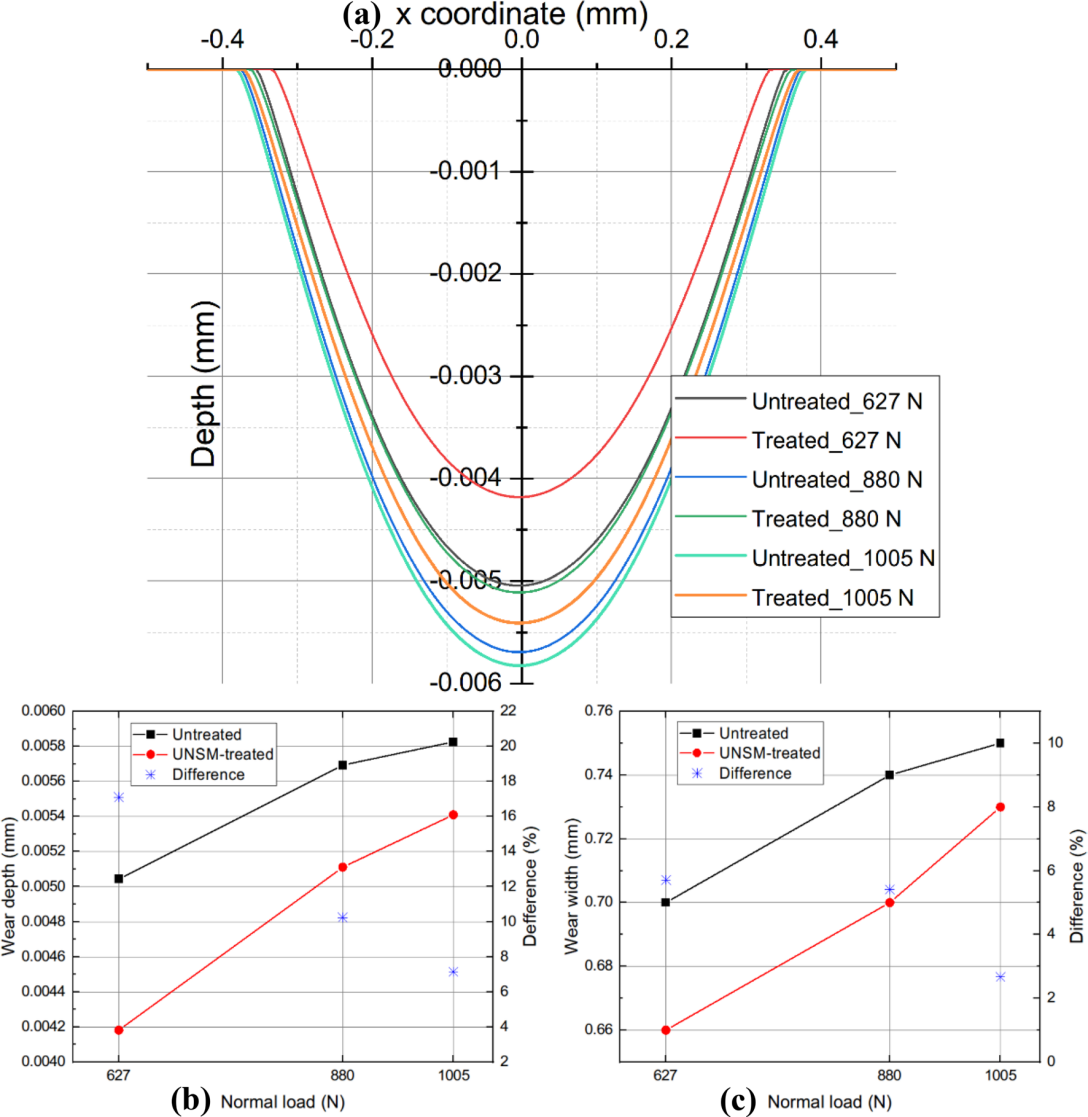
Comparison of wear profiles (**a**), wear depth (**b**), and wear width (**c**) for untreated and UNSM-treated alloy 718 with modified wear coefficient under various normal loads.

FEM can be used to observe the effect of the UNSM-optimized wear coefficient on the wear performance of Cases 1.2, 3.2, and 5.2. According to Eq. (7), the wear coefficient plays a crucial role in directly reducing the surface's wearability. The UNSM-modified wear coefficient results in a lower incremental wear depth, $\it \Delta y$, in a unit stress state and a relative sliding distance than the unmodified coefficient. As a result, the wear scar eventually becomes weaker, as depicted in Fig. 14. When *P* = 627 N, the wear depth is decreased by 17% and the wear width is reduced by 5.7%. However, as *P* increases, the degree of relief provided by the UNSM-optimized wear coefficient diminishes gradually. When *P* reaches 1005 N, the UNSM-optimized wear depth and width mitigation decreases to 7.14% and 2.67%, respectively.

**Figure 15:**
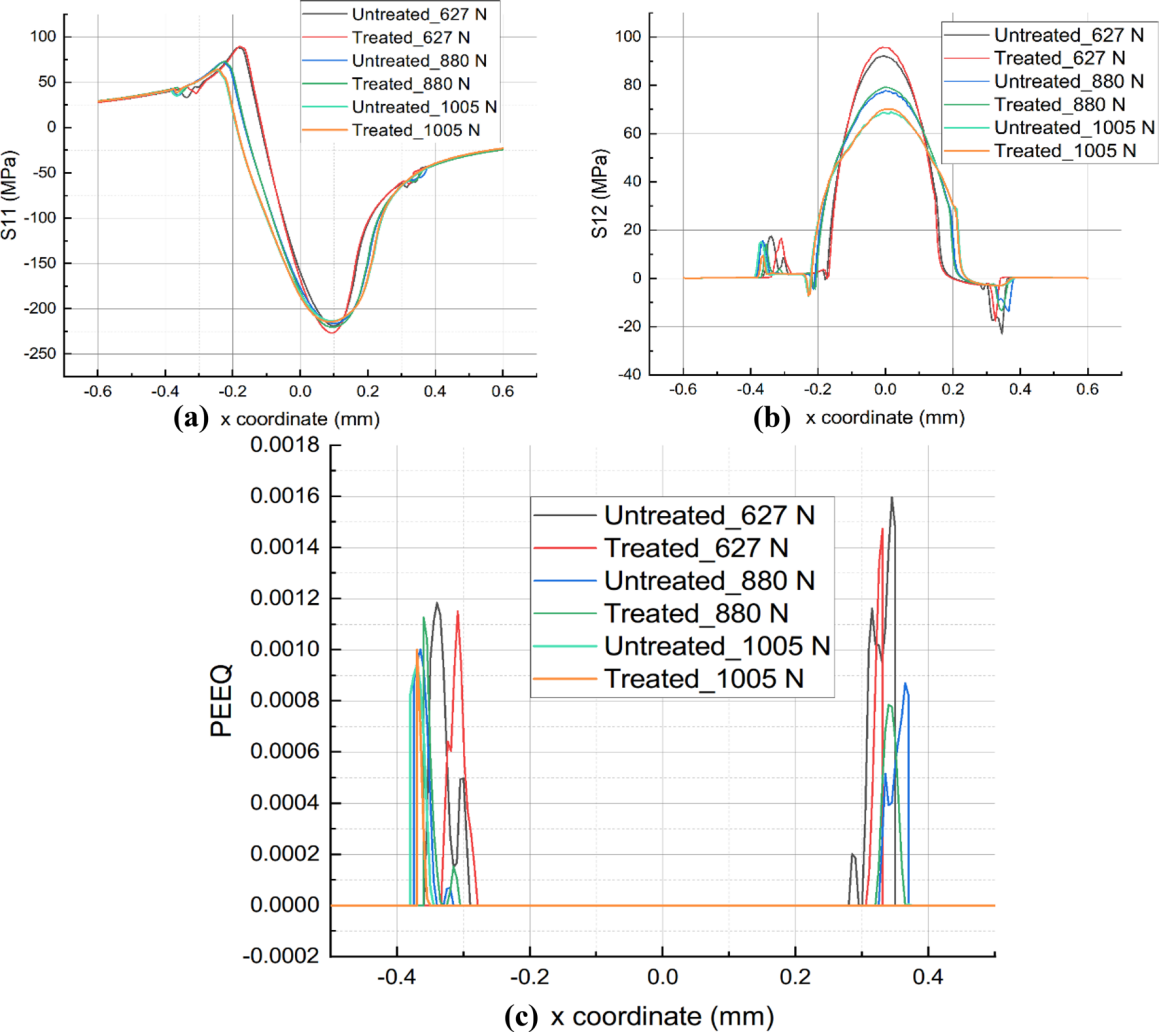
Comparison of S11 (**a**), S12 (**b**), and PEEQ (**c**) for as-printed and UNSM-treated alloy 718 with modified wear coefficient under various normal loads.

As shown in Figs. 15a and 15b, the modified wear coefficient hardly affects the stress distribution, especially for S11. Since the objective is to examine the influence of the UNSM-modified wear coefficient on the wear performance, the COF is fixed under the same normal load *P* for each sample. For instance, when *P* = 627 N, the magnitude of S11 is nearly identical, with a variance of approximately 1.32%, and exhibits the same regularity at other normal loads *P*. Regarding S12, the optimized wear coefficient results in a greater amplitude at the same load *P*. The reason for this is that the wear scar considering the UNSM-modified wear coefficient is weaker than that of the untreated surface, indicating a smaller contact area during the previous cycle. Consequently, the contact pressure will increase and S12 will increase for the same COF value. Notably, the UNSM-optimized wear coefficient alone has little effect on the distribution of S12 as *P* increases to 1005 N.

As shown in Fig. 15c, the distribution of PEEQ exhibits a canyon-like pattern. Due to the high cyclic fretting wear, the middle region of the surface is largely depleted of material, leaving only the elastic region with zero plastic strain. At present, the plastic strain is more easily concentrated at the edge of the wear scar. This is due to the high geometric gradient at the edge of the wear scar, which causes a higher stress state and, consequently, a more rapid accumulation of plastic strain. The UNSM-modified wear coefficient also results in a narrower wear scar. Consequently, under the same normal load *P*, the PEEQ gradually moves inward as the scar diminishes as it contracts.

## Conclusions

In this work, the alloy 718 samples are fabricated by DED from Inconel 718 alloy powder and then processed by wire cutting system. Due to the poor surface qualities, UNSM technology is used to modify the surface properties of alloy 718. To study the impact of UNSM on wear performance, a finite element model using dissipated energy method is developed. The conclusion is given as follows:

- The DED is used to prepare the alloy 718 samples, which are then processed using UNSM technology. The uniaxial tensile test shows that the UNSM can significantly improve the mechanical properties of alloy 718. A fretting wear testing setup based on the cylinder-on-plane configuration is constructed, which is conducted on untreated and UNSM-treated 718 alloy under three high normal loads *P*, namely 627 N, 880 N, and 1005 N. Moreover, the isotropic hardening law is incorporated into the constitutive model to capture the plastic behavior under high normal load *P*.
- The finite element results exhibit good agreement with the experimental measurements, and the error of wear volume is within 2% after 20,000 cycles. In addition, the simulation results show that UNSM can effectively enhance the wear resistance of alloy 718 for various normal loads. When *P* = 627 N, the relief degree of wear depth reaches 24.22% compared with the untreated one. However, the lift in wear performance of UNSM at high normal loads *P* is not significant. When *P* = 1005 N, the mitigation induced by UNSM on wear depth drops to 2.82%.
- Not only the overall effect of UNSM on wear performance, but also the effect of each parameter on wear behavior is discussed separately. Since the energy-based fretting wear model is employed in this study, the UNSM technology primarily influences the incremental wear depth by modifying the COF and energy wear coefficient. Taking *P* = 627 N as an example, the UNSM-modified COF reduces wear depth by 8.89% compared to the untreated sample. Additionally, the UNSM-optimized wear coefficient excels in wear resistance and decreases the wear depth by 17%. By reducing the COF along the surface, UNSM technology weakens the distribution of shear stress S12 in finer detail. Therefore, it diminishes the incremental wear depth according to the energy-based wear method. By combining the modified COF and wear coefficient at a lower normal load *P*, UNSM can significantly improve the wear resistance. However, as the normal load *P* rises, the UNSM-optimized COF and wear coefficient contribute less to fretting wear mitigation.

## Data Availability

The datasets used and/or analyzed during the current study available from the corresponding author on reasonable request.
